# Fruit and vegetable consumption across population segments: evidence from a national household survey

**DOI:** 10.1186/s41043-023-00382-6

**Published:** 2023-06-08

**Authors:** Nihat Küçük, Faruk Urak, Abdulbaki Bilgic, Wojciech J. Florkowski, Adiqa K. Kiani, Ferda Nur Özdemir

**Affiliations:** 1 Department of Economics, College of Economics and Administrative Sciences, Harran University, Osmanbey Campus, 2nd Floor, Sanliurfa, Turkey; 2 TRT Erzurum Regional Directorate, The Turkish Radio and Television Corporation (TRT), Adnan Menderes Mahallesi, Fatih Sultan Mehmet Cd., Room # 5, Erzurum, Turkey; 3 Department of Management Information Systems, College of Economics and Administrative Sciences, Bilecik Seyh Edebali University, A Block 3rd Floor, Room #323, Bilecik, Turkey; 4 Department of Agricultural and Applied Economics, The University of Georgia, 1109 Experiment Street, Griffin, GA 30223 USA; 5 Future Technology Research Centre, National Yunlin University of Science and Technology, Douliu, Taiwan; 6 Department of Agricultural Economics, Faculty of Agriculture, Atatürk University, Dean’s Building, 2nd Floor, Room #17, Erzurum, Turkey

**Keywords:** Fruits and vegetable intake, Individual characteristics, Households, Obese, Income, Walking, Intra-familial heterogeneity, Multilevel data, Random-effects bivariate probit, Turkey

## Abstract

**Background:**

The 2002 World Health Report documented that low fruit and vegetable intake are among the top ten risk factors contributing to attributable mortality and up to three million lives could be saved each year by adequate consumption of F&V across the globe, leading an examination of behavioral preferences of the individual and family social, environmental, and behavioral factors that constitute perceived barriers to fruit and vegetable consumption.

**Objective:**

The study examines factors affecting the choice of eating fruits and vegetables by household members and calculates eating frequency probabilities of different population-origin associated with personal attributes and behavior.

**Method:**

Turkish Health Survey (THS) 2019 data from the Turkish Statistical Institute (TSI) national representative household panel is applied. Estimating a random-effect bivariate probit model of fruit and vegetable choice, we calculated marginal probabilities of choosing fruits and vegetables, the joint probability of choosing both, and conditional probabilities between choosing to eat either, detecting consumption synergy.

**Results:**

The role of uncontrolled variables in choosing to eat fruits and vegetable (F&V) differs between the decision of an average family and the decision of individual family members. The attitude is positive for an average family and contrasts with the negative attitude among some family members. Most individual and family attributes inversely affect fruit and vegetable choice across different groups, while a positive relationship exists between the likelihood of fruit and vegetable choice and attributes such as age, marital status, education, weight, having health insurance, income, and time and forms of physical activity.

**Conclusion and recommendation:**

Instead of a general policy for the implementation of a healthy and balanced nutrition program to improve fruit and vegetable eating frequency, it appears more effective to adopt programs with distinct characteristics that segregate society into different cohorts. We suggest appropriate policies and offer suitable approaches to reach targeted groups.

## Introduction

The 2002 World Health Report documented that low fruit and vegetable intake are among the top ten risk factors contributing to attributable mortality and up to three million lives could be saved each year by adequate consumption of F&V across the globe [1]. The daily, habitual intake of F&V can prevent major non-communicable diseases (NCDs) (some cancers and cardiovascular diseases) while eating a variety of produce daily provides the body with the most micronutrients, dietary fiber, and essential non-nutrients [2]. F&V, rich in phytochemicals such as polyphenols, terpenoids, and organic sulfides, play a role in adipose tissue growth and differentiation, apoptosis of adipocytes, and lipid and energy metabolism [3]. About 85% of the global disease burden attributable to low fruit and vegetable intake (LFVI) includes 31% of ischemic heart disease, 15% of cancers, and 11% of strokes [4]. Also, inadequate consumption of F&V causes 7.5% (from lack of fruits) and 7.6% (from lack of vegetables) of deaths from heart disease, stroke, and type 2 diabetes [5] While lowering food and beverage consumption provides a significant fall in both body mass index (BMI) [6] and regulates blood pressure (BP) and blood sugar [7], increased food and beverage consumption can alleviate the risk of depression [8] with more robust mental health [8] and provide positive psychological effects on humans [9]. Several countries, therefore, implemented public education campaigns to increase F&V consumption. Campaigns in Europe and North America have increased F&V consumption. The American National Academy of Sciences urged a change in F&V consumption habits of low-income families using the “Five a Day” program, which has been well-received over the past two decades [10].

Although most chronic diseases can be prevented by lifestyle choices such as healthy eating and physical activity [5, 10], in past studies, fruit and vegetable consumption decisions have been limited to socio-demographic, economic, and behavioral factors. Specific traits included gender, age, and education, while other characteristics accounted for psychological, behavioral, and biological factors [11, 12]. Measures of income and location complete the list. The majority of empirical studies focus on individual behavior, not accounting for a family, and therefore are difficult to implement in settings where individual decisions about fruit and vegetable consumption are made at the family level. Studies are lacking on the effects of factors on the fruit and vegetable consumption decisions of individuals within a family.

An examination of behavioral preferences of the individual and family social, environmental, and behavioral factors that constitute perceived barriers to fruit and vegetable consumption is needed. Identifying characteristics that sustain balanced, daily F&V consumption in emerging economies such as Turkey could increase inadequate intake and reduce the risk factors contributing to attributable mortality. The current study, therefore, aims to determine the empirical heterogeneity of the relationship between fruit and vegetable intake and individual and family traits using a random-effect bivariate probit model. The study uses nationally representative intra-family multi-level data from Turkey. The bivariate random-effects model tests the presence and strength of factors after controlling for within-family observable traits in each consumption event. This study, first in applying a simultaneous approach of both food types to F&V consumption, contributes to the existing literature by aiming to control the intra-familial heterogeneity. Considering the heterogeneity in dietary habits and food culture of individuals within the family and understanding what influences dietary choices in F&V consumption is crucial for the development of effective interventions leading to sustainable behavioral change. Increased F&V consumption is supported by the well-established link between F&V consumption, health maintenance, and disease prevention and is of great concern to policymakers. The benefits from higher F&V consumption are not limited to individuals but lower the costs of health services by public and private providers. As Turkey’s population grows, the potential cost savings could be substantial.

The current study also provides extensive empirical insights for food industry managers to create effective and dynamic marketing strategies. Food distributors and retailers can offer consumer nutritional education programs using knowledge of distinct individual profiles within a family generated from the current study. Formulating marketing strategies and nutritional programs tailored to consumer consumption patterns have the potential to be an effective intervention tool promoting greater fruit and vegetable intake. The current study provides robust information about diverse fruit and vegetable consumption, for example, the habits of those who consume fruits and vegetables separately (measured by marginal probability), those who consume both together (i.e., quantifying joint probability), and those who consume fruit (vegetables) when also consuming vegetables (fruit) (measured by conditional probability). In this context, information obtained from different consumption probability components will provide policymakers with qualified insights for more efficient use (e.g., balanced choice of nutrients) and distribution of national resources. Also, inferences derived from the current study facilitate the implementation of comparable actions in other countries with similar consumer socio-demographic, economic, and eating pattern characteristics as in Turkey.

### Fruit and vegetable consumption and nutrient deficiency in Turkey

In Turkey, fruit and vegetable consumption follows the pattern of several major emerging and developed economies. The annual F&V consumption in Turkey was 32.87/122.33 kg, while in Brazil, Canada, China, France, Greece, England, and the United States was 19.97/26.97 kg, 29.65/71.43 kg, 36.77/330.68 kg, 30.98/65.06 kg, 53.15/77.13 kg, 22.23/55.93 kg, and 20.15/65.98 kg, respectively, in 2019 [13]. Per-capita fruit and vegetable consumption in Turkey is high when compared to other listed countries. The daily energy intake of fruits and vegetables per capita in Brazil, Canada, China, France, Greece, Turkey, England, and the USA, was 20/18 kcal, 44/58 kcal, 42/225 kcal, 46/52 kcal, 69/49 kcal, 41/70 kcal, 36/46 kcal, and 31/44 kcal, respectively [13]. Turkey’s per capita daily fruit and vegetable energy intake was also higher than the other listed countries. However, the Turkish population suffers from nutrient deficiencies similar to other countries. The deficiency involves several key vitamins and micronutrients essential for healthy, productive living. Vitamin A deficiency, which also affects iron levels, was found among 17% of pregnant women in Turkey [14]. Tomatoes, green peppers, and cucumbers that provide vitamin A are commonly grown and eaten vegetables in Turkey. Turkey was the world’s third-largest tomato producer in 2019 [15].

Consumption of F&V offsets the lack of knowledge about the importance of folic acid among Turkish consumers, especially during pregnancy [16], and is particularly important for those who do not take folic acid-containing supplements. Increased folic acid is needed to reduce the incidence of neural tube defects in Turkey [17]. Leafy greens, broccoli, and asparagus, among vegetables, and citrus fruit and bananas, among fruits, are valuable sources of folic acid. Citrus and bananas are domestically produced and accessible. Among micronutrients, iron deficiency has been verified among reproductive-age women in a study on anemia in Turkey. Iron deficiency is preventable at a relatively low cost and through targeted education campaigns [18]. Such a campaign could publicize that eating domestically produced fruits (e.g., citrus, banana) and vegetables (e.g., tomatoes, green peppers, and cucumbers,) supplies the body with iron (Table 4).

Hypokalemia is a new phenomenon associated with the inadequate intake of potassium resulting from a decreased level of potassium in the soil where crops grow [19]. A comparison of the potassium content of fruits and vegetables in the United States between 1999 and 2015 showed a decrease in the case of several fruits and vegetables commonly consumed in Turkey. For example, among fruits, lower potassium content was verified in apples, apricots, bananas, figs, kiwis, melons, and watermelons. Among vegetables, decreased potassium content was reported in broccoli, cabbage, cauliflower, onions, peppers, and potatoes, among others. Turkey was the fifth producer of onions in the world in 2019 [20]. Turkish consumers have been more knowledgeable about dietary fiber consumption than consumers in several other countries [21].

A study of Turkish consumers reported that the average vegetable consumption for four age groups was about once per day, except that in the case of the elderly the average was slightly above (1.6 consumption occurrences per day) [21]. Fruit consumption was considerably higher and increased from 1.4 eating occurrences per day for young adults to 2 eating occasions for the elderly. Eggplant, green peppers, and yellow onions are good sources of fiber among the popular vegetables in Turkey (Table 5). Among fruits that are produced in Turkey, citrus, banana, and apples provide even more fiber per serving than most vegetables (Table 4). Bananas and apples also provide small amounts to vitamin B_12_, not reported sources used to create Table 4. Turkey is among the leading apple-producing countries in the world [20].

The limited consumption of fruits and vegetables as a source of vitamins and key nutrients contrasts with the recent surge in internet searches about vitamins exacerbated by the COVID-19 epidemic [22]. Often the bioavailability of key nutrients in F&V surpasses that of supplements [23], although the issue is complex and requires additional research [24]. In the meantime, Turkish consumers can enjoy the abundant domestic supply of fresh produce and benefit from the established link between its consumption and disease prevention and health maintenance.

## Materials and methods

The cross-sectional data used in the study was compiled from Turkey Health Survey (THS) 2019. The survey, conducted by the Turkish Statistical Institute (TSI), has been carried out biennially since 2008 in cooperation with the Statistical Office of the European Union (SOEU). The surveys are conducted in the last quarter of the year (October, November, and December), and the total number of observations is determined as per the survey modules of the SOEU. The total sample size was 9470 household addresses and 8166 families (i.e., 88% participation rate) interviewed. The 1304 non-participating households include either those who accepted the administrative fine or those who were not present during the survey (e.g., due to vacation or visiting relatives). The participants were 18 years old or older. Twelve statistical subdivisions of the country were included in the Nomenclature of Territorial Units for Statistics (NUTS).

Survey information about the respondent characteristics includes gender, age, marital status, education, employment status, body mass index, health insurance, participation in physical exercise, leisure, tobacco and alcohol use, time allocated to walking and other types of physical exercise, occupation, history of depression,^1^ and the number of chronic diseases, among others. A separate set of questions asked about family traits such as the number of children in different age groups (under 7; 7 to 14), number of adults, income categories, and residential location. Several other questions were about education, age, and income.

Our empirical specification in the current study is laid down from the discrete random utility theory [25], in which a household maximizes its random utility function subject to a fixed budget:

1 $$\mathop {\max }\limits_{q,c} \{ U\left( {Yq,c;h} \right)|p^{\prime}q + c = m\}$$

where $q=[{q}_{1},{q}_{2}]{^{\prime}}$ is a vector containing quantities of fruits $({q}_{1})$ and vegetables $({q}_{2})$ with their corresponding unit prices $p = [p_{1} ,p_{2} ]^{\prime }$, respectively. While c is a composite commodity for all but these two food products, *m* reflects the budget, and *h* is a vector of the household, but mostly characteristics of an individual. While we set $Y=diag\left({y}_{1},{y}_{2}\right)$ as a diagonal matrix with each (random) binary indicator ${y}_{i}$ indicating potential consumption of ${q}_{i}$ [26]. Assuming that the utility function U(Y*q,c;h*) is strictly quasi-concave and increases with increasing positive values of *Yq* and *c* leads to the notional demand of the two foods. Such notional demands are obtained by solving the utility maximization problem in Eq. (1), in which a vector of optimal quantities demanded of both foods is a function of their prices without non-negativity constraints, household budget, as well as characteristics of individuals [26]. The use of individuals and household characteristics in demand analysis with cross-sectional data dates back to [27].

Our dependent variables are consumption probabilities, as we only observe how often these two foods are consumed daily, not their quantities in a household. If the individual consumes one or more servings of fruit or vegetables daily, we defined them as the latent variables (y_1_ for fruits and y_2_ for vegetables). The random-effects bivariate probit model consists of households (i = 1,…,N), two heterogeneity parameters, α_1_, and α_2_, defined for the family members (j = 1,…,M) by the exogenous variables **x**_1_ and **x**_2_, and possibly associated error terms ε_1_ and ε_2_, unit variances, correlation coefficient (τ), and two latent variables, y_1_, and y_2_, that are normally distributed:

2 $$\begin{aligned} y_{1,ij}^{*} & = x^{\prime}_{1,ij} \beta_{1} + \alpha_{1,i} + \varepsilon_{1,ij} \\ y_{2,ij}^{*} & = x^{\prime}_{2,ij} \beta_{2} + \alpha_{2,i} + \varepsilon_{2,ij} ,\,\,\,\,\,i = 1,...,N\,\,and\,\,j = 1,...,M \\ \end{aligned}$$

where

3 $$\begin{aligned} \varepsilon_{it} & = \left( \begin{gathered} \varepsilon_{1,ij} \hfill \\ \varepsilon_{2,ij} \hfill \\ \end{gathered} \right) \approx i.i.d.N\left[ {\left( \begin{gathered} 0 \hfill \\ 0 \hfill \\ \end{gathered} \right);\left( \begin{gathered} 1\,\,\,\,\tau \hfill \\ \tau \,\,\,\,1 \hfill \\ \end{gathered} \right)} \right] \\ \alpha_{i} & = \left( \begin{gathered} \alpha_{1,i} \hfill \\ \alpha_{2,i} \hfill \\ \end{gathered} \right) \approx i.i.d.N\left[ {\left( \begin{gathered} 0 \hfill \\ 0 \hfill \\ \end{gathered} \right);\left[ \begin{gathered} \sigma_{1}^{2} \,\,\,\,\,\,\,\,\,\,\,\rho \sigma_{1} \sigma_{2} \hfill \\ \rho \sigma_{1} \sigma_{2} \,\,\,\,\,\,\,\,\sigma_{2}^{2} \,\, \hfill \\ \end{gathered} \right]} \right] \\ \end{aligned}$$

The coefficient τ is the degree of relationship between two latent variables (y_1_ and y_1_), coefficients σ_i_ denote the associated standard deviations of the two heterogeneous coefficients (α_i_), ρ represents the correlation coefficient between the two heterogeneous coefficients, while β_i_ denotes the coefficients of regressor variables affecting the latent variable. In our example, the fixed-effects model (in its most basic form) may control for unmeasured variables that are constant between individuals but vary between families, by explicitly including a separate cutoff term (α_i_) for each family in each regression equation above. However, the reason for using the random-effects model instead of the fixed-effects model is that, in addition to the confounding variables originating from the family, we cannot fully predict how all confounding factors specific to individuals (such as the individual’s talent, ability, digestion, appetite, other health factors, etc.) will react from family to family and the probability of fruit and vegetable consumption. The same is true for mixed-effects. In addition, the concern that many of our control variables might have collinear relationships with individual unmeasured or confounding variables led us to the random-effects model. Our random-effects model is also equivalent to the random parameter model, with only the constant term being random. Although the triggers (regressors) of each latent variable could be different, they are assumed to be equal in this study (**x**_1_ = **x**_2_). The observed model is:

4 $$\begin{aligned} y_{1,ij} & = 1\,\,(y_{1,ij}^{*} > 0) \\ y_{2,ij} & = 1\,\,(y_{2,ij}^{*} > 0) \\ \end{aligned}$$

where y_1_ is 1 if an individual consumes fruits one or more servings (portions) per day, 0 otherwise. Similarly, y_2_ is 1 if an individual consumes vegetables one or more servings per day, and 0 otherwise. In other words, it reflects that the daily intake of at least one or more servings of fruits and vegetables is related to the characteristics of the individual and the family.

The classical transformation of the observed variables and the corresponding conditional composite likelihood functions are defined, respectively, as^2^:

5 $$\begin{aligned} q_{1,ij} & = 2y_{1,ij} - 1 \\ q_{2,ij} & = 2y_{2,ij} - 1 \\ \ell_{i} (y_{i} |x_{ij} ,\beta ,\sigma_{i}^{2} ,\rho ) & = \int\limits_{ - \infty }^{ + \infty } {\int\limits_{ - \infty }^{ + \infty } {f_{i} (y_{i} |x_{i} ,\alpha_{i} ,\beta ,\tau )} } *g\left( {\alpha_{i} |\sigma_{1}^{2} ,\sigma_{1}^{2} ,\rho } \right)d\alpha_{1,i} d\alpha_{2,i} \\ \end{aligned}$$

While some studies took advantage of simulations to optimize the above likelihood function [28], this study employs the Gauss-Hermite quadratic technique [29]. The random-effects bivariate probit model is essentially identical to the bivariate random parameter probit model when the constant coefficient is assumed to be random.^3^

Probability values are derived differently when using a random-effects bivariate probit model. For example, besides examining the probability of consuming fruits and vegetables together (e.g., the joint decision), it is possible to obtain the conditional consumption probability of fruits (vegetables) given the consumption probability of vegetables (fruits). The expected values of marginal (e.g., individual), joint, and conditional probabilities of F&V intake are:

6 $$\begin{aligned} \Pr \left[ {y_{k,ij} = 1|x_{k,ij} } \right] & = \Phi \left( {x^{\prime}_{k,ij} \hat{w}_{k} } \right),\,\,k = 1,2 \\ \Pr \left[ {y_{1,ij} = 1,y_{2,ij} = 1|x_{k,ij} } \right] & = \Phi_{2} \left( {x^{\prime}_{1,ij} \hat{w}_{1} ,x^{\prime}_{2,ij} \hat{w}_{2} ;\tau } \right) \\ \Pr \left[ {y_{k,ij} = 1|y_{m,ij} = 1} \right] & = \frac{{\Pr \left[ {y_{1,ij} = 1,y_{2,ij} = 1|x_{k,ij} } \right]}}{{\Pr \left[ {y_{m,ij} = 1|x_{k,ij} } \right]}},\,\,k \ne m \\ \end{aligned}$$

where $\hat{w}_{k} = \frac{{\hat{\beta }_{k} }}{{\sqrt {1 + \hat{\sigma }_{k}^{2} } }}$ and $\Phi \,$ and Φ_2_ are univariate and bivariate cumulative density functions, respectively. Differentiation of each corresponding probability value concerning any regressor is the marginal (unitary) effect in the case of a continuous variable and a unitary effect if the explanatory variable is binary. The standard deviations of marginal effects were obtained using the delta method.

## Descriptive and preliminary results

Table 1 shows the descriptive statistics of dependent and confounding variables. The consumption rates of one or more servings per day were 47% for fruits and 57% for vegetables. Daily fruit and vegetable consumption is common, with nearly one in two individuals eating one or more servings per day. Considering that in Turkish culture, meals, especially dinners, are served in the presence of the whole family with a vegetable salad served first, a high vegetable intake is expected. Fruit intake, on the other hand, varies according to the individual’s preferences, taste, and psychological state at the time it is offered. Approximately 40% of individuals consume both foods together (Table 3). About 70% of those who consume one or more servings of vegetables a day get fruit, while about 85% of those who consume one or more servings of fruit a day consume vegetables. Among those who consume vegetables, the habit of consuming fruit is weaker than the habit of consuming vegetables among those who consume fruits. In addition, the intake of both foods is at a very low rate. The fact that those who consume fruits are more conscious in terms of health also increases the odds of consuming vegetables. However, to boost fruit intake among those who consume vegetables and increase consumption of both foods together as a part of daily life in individuals, there is a need for intervention initiatives in the country.

**Table 1:** Means and VIF scores of dependent and explanatory variables

Variables	Descriptive	Mean (SD)	VIF
*Dependent variables*			
Fruit intake	1if eating fruits one or more servings per day, 0 otherwise	0.473 (0.499)	–
Vegetable intake	1if eating vegetables one or more servings per day, 0 otherwise	0.571 (0.495)	–
*Independent variables: (0/1) Dummy variables*			
Gender	1 if male, 0 otherwise	0.456 (0.498)	1.898
Married	1 if married, 0 otherwise	0.686 (0.464)	1.468
No school	1 if no school, 0 otherwise (reference group)	0.128 (0.335)	–
Elementary school	1 if elementary school diploma,0 otherwise	0.329 (0.470)	2.532
Secondary school	1 if secondary school diploma,0 otherwise	0.174 (0.379)	2.253
High school	1 if high school diploma,0 otherwise	0.190 (0.392)	2.308
Community college	1 if a two-year community college, 0 otherwise	0.055 (0.227)	1.538
College	1 if college degree including post-graduate, 0 otherwise	0.125 (0.331)	2.295
Student & Military	1 if the individual is a student or in military service, 0 otherwise	0.076 (0.265)	–
Wage Job	1 if the person is paid, 0 otherwise	0.287 (0.453)	4.817
Employer	1 if the individual is the employer, 0 otherwise	0.095 (0.294)	2.859
Job seekers	1 if the person seeks a job, 0 otherwise	0.059 (0.235)	1.855
Retired	1 if retired, 0 otherwise	0.143 (0.350)	4.721
Homemaker	1 if the person works as a homemaker, 0 otherwise	0.340 (0.474)	5.738
Normal weight	1 if the individual has a normal weight, 0 otherwise (reference group)	0.419 (0.494)	–
Overweight	1 if BMI > 25and BM I ≤ 30, 0 otherwise	0.358 (0.479)	1.323
Obese	1 if BMI > 30and BMI ≤ 35, 0 otherwise	0.165 (0.371)	1.305
Morbidly obese	1 if BMI > 35, 0 otherwise	0.058 (0.235)	1.156
General health insurance	1 if general health coverage, 0 otherwise	0.921 (0.270)	0.946
Private health insurance	1 if pays health expenses out of pocket, 0 otherwise	0.037 (0.190)	1.125
Cycling	1 if cycling for 10 min at least a day a week, 0 otherwise	0.171 (0.376)	1.054
Walking < 10 min	1 if the person walks less than 10 min on a normal day, 0 otherwise	0.168 (0.374)	–
Walking 10–29 min	1 if the person walks less than 10–29 min on a normal day, 0 otherwise	0.392 (0.488)	2.010
Walking 30–59 min	1 if the person walks less than 30–59 min on a normal day, 0 otherwise	0.269 (0.444)	1.949
Walking 1–2 h	1 if a person walks for 1–2 h on a normal day, 0 otherwise	0.118 (0.323)	1.566
Walking > 2 h	1 if the person walks for more than 2 h on a normal day, 0 otherwise	0.050 (0.218)	1.291
Resting	1 if the person sits and rests less than 4 h is a day, 0 otherwise	0.356 (0.479)	1.085
Light physical job	1 if the person works in a mostly sitting or standing job, 0 otherwise (reference group)	0.636 (0.481)	–
Moderate physical job	1 if the person works in a job that often requires walking or moderate physical strength, 0 otherwise	0.323 (0.468)	1.146
Demanding physical job	1 if the person works in jobs that require heavy work or physical strength, 0 otherwise	0.040 (0.196)	1.131
Low income	1 household income less than 992₺, 0 otherwise	0.047 (0.211)	1.058
Middle income	1 household income between 992–8913₺, 0 otherwise (reference group)	0.898 (0.303)	–
High income	1 household income greater than 8913 ₺, 0 otherwise	0.056 (0.229)	1.153
Eastern Anatolia	1 the Eastern Anatolia resident, 0 otherwise (reference group)	0.072 (0.259)	–
Marmara	1 if Marmara resident, 0 otherwise	0.284 (0.451)	3.164
Aegean	1 if Central Aegean resident, 0 otherwise	0.055 (0.228)	1.575
Mediterranean	1 if Mediterranean region resident, 0 otherwise	0.101 (0.301)	2.027
Black Sea	1 if Black Sea region resident, 0 otherwise	0.284 (0.451)	3.144
Central Anatolia	1 if Central Anatolia region resident, 0 otherwise	0.161 (0.368)	2.469
Southeastern Anatolia	1 if Southeastern Anatolia region resident, 0 otherwise	0.042 (0.201)	1.447
*Independent variables: Continuous variables*			
Age	Age of the person in years	43.954 (17.673)	2.441
Sports Time	Time devoting sports on a day	0.264 (1.355)	1.054
Tobacco	Amount of packs used per day	0.011 (0.022)	1.193
Alcohol	Number of glasses used per day	1.800 (3.159)	1.246
Number of children under 7	The number of children between the ages of 0–6	0.343 (0.674)	1.248
Number of kids ages 7–14	The number of children between the ages of 7–14	0.434 (0.767)	1.113
Number of adults	The number of persons 15 years or older	2.561 (1.137)	0.963
# of individuals	17,084		
# of families	8166		

The individuals participating in the survey (Table 1) included 45.5% male, 54.5% female, 68.6% married, about 18% university graduates, 16% obese as well as 6% extremely obese, 46% smokers, and 5% consuming alcohol. Further, 3% participated in weight lifting at least four times a week, and 16% engaged in sports for at least one hour a day. Descriptive statistics of other variables are given in Table 1. The variance inflation factor (VIF) was calculated to verify the absence of multicollinearity among the independent variables, where a VIF value of less than 5 indicates that there is no significant collinear relationship between our regressors in this study.

Table 2 shows the estimators of the maximum likelihood function. The correlation coefficient (τ) between the two consumption decisions was positive and statistically significant (Table 2). While vegetables are mostly used within meals and served in the form of salads, it is a classic Turkish tradition to serve fruit immediately after a meal or a few hours after dinner. Therefore, when uncontrollable factors affect the vegetable intake decision, they also likely affect the probability of fruit intake. The correlation coefficient (ρ) between heterogeneities in the consumption probabilities of F&V was negative and statistically significant. The heterogeneity, for example, in the consumption probability of fruits has an inverse effect on the heterogeneity of vegetable consumption. Interestingly, while the two food intake patterns are positively affected by uncontrollable variables among families, they are negatively affected among family members. This is a very important result because the average attitudes of family members may differ from those of the average family. The Likelihood Ratio (LR) test (LR = −4051.87, degrees of freedom (df) = 2, *p* < 0.000) rejected the hypothesis that both correlation coefficients were simultaneously zero, indicating that the bivariate random-effects probit model was superior to the two discrete (e.g., separate) binary random-effects probit models. Also, using the Lagrangian Multiplier (LM) test (LM = 3923.08, df = 4, *p* < 0.000), the goodness of fit of the proposed bivariate-random effects probit model outperforms the bivariate probit model,^4^ which ignores all intra-family heterogeneities. Using the LR test, we also determined that all regressors used in the random-effects bivariate probit model are the main source of variation in the probability of simultaneous consumption decision (LR = 80.00, df = 80, *p* < 0.000). The following discussion will focus on the proposed model. Also, the overlap between the actual values and the estimated values in all calculated probabilities is strong evidence of the suitability of the method used in the data analysis (Table 3).

**Table 2:** Maximum likelihood estimates from the panel random-effects bivariate probit model

Variables	Fruits		Vegetables	
	Coefficient	Standard Error	Coefficient	Standard Error
Constant	−0.812***	0.103	−0.326***	0.111
*Discrete variables*				
Gender	−0.220***	0.031	−0.451***	0.035
Married	0.089***	0.029	0.191***	0.032
Elementary school	0.304***	0.044	0.398***	0.049
Secondary school	0.479***	0.053	0.448***	0.057
High school	0.386***	0.051	0.396***	0.055
Community college	0.561***	0.067	0.551***	0.072
College	0.426***	0.059	0.445***	0.063
Wage Job	0.034	0.053	0.049	0.056
Employer	−0.041	0.063	0.112*	0.068
Job seekers	−0.079	0.065	0.020	0.070
Retired	0.158**	0.070	0.107	0.077
Homemaker	−0.009	0.057	−0.022	0.062
Overweight	0.013	0.027	0.001	0.030
Obese	0.141***	0.036	0.036	0.039
Morbidly obese	0.126**	0.056	0.133**	0.058
General health insurance	0.082**	0.041	0.024	0.044
Private health insurance	0.052	0.066	0.090	0.069
Cycling	0.197***	0.049	0.023	0.054
Walking 10–29 min	0.026	0.034	0.131***	0.038
Walking 30–59 min	0.102***	0.037	0.225***	0.040
Walking 1–2 h	0.089*	0.046	0.223***	0.049
Walking > 2 h	0.156***	0.059	0.254***	0.067
Resting	0.029	0.024	0.078***	0.027
Moderate physical job	0.063**	0.025	0.108***	0.027
Heavy physical job	0.101*	0.057	0.107*	0.063
Low income	−0.428***	0.056	−0.706***	0.061
High income	0.162***	0.053	0.414***	0.058
Marmara	0.102**	0.048	0.026	0.053
Aegean	−0.390***	0.065	−0.460***	0.071
Mediterranean	−0.421***	0.056	−0.378***	0.061
Black Sea	−0.672***	0.049	−0.934***	0.054
Central Anatolia	−0.020	0.051	−0.054	0.057
Southeastern Anatolia	−0.660***	0.071	−0.928***	0.078
*Continuous variables*				
Age	0.011***	0.001	0.010***	0.001
Sports Time	0.049***	0.008	0.039***	0.010
Tobacco	−0.297***	0.027	−0.108***	0.008
Alcohol	−0.011***	0.004	−0.006	0.029
Number of children under 7	−0.005	0.019	−0.068***	0.004
Number of kids ages 7–14	−0.074***	0.016	−0.048***	0.020
Number of adults	0.001	0.009	0.007	0.018
σ^2^	1.257***	0.017	1.115	0.017
ρ	−0.749***	0.018	–	–
τ	0.697***	0.011	–	–

***p < 0.01, **p < 0.05, *p < 0.10

**Table 3:** Marginal effects of explanatory variables on eating one or more servings of fruits and vegetables in Turkey using the bivariate random-effects probit model

Variables	Prob(y1 = 1)		Prob(y2 = 1)		Prob (y1 = 1, y2 = 1)		Prob (y1 = 1|y2 = 1)		Prob (y2 = 1|y1 = 1)	
	ME*100	Std. Err	ME*100	Std. Err	ME*100	Std. Err	ME*100	Std. Err	ME*100	Std. Err
*Discrete variables*										
Gender	−5.441***	0.765	−11.827***	0.905	−7.911***	0.685	−0.043	0.841	−7.422***	0.710
Married	2.196***	0.722	5.015***	0.838	3.284***	0.636	−0.106	0.785	3.194***	0.655
Elementary school	7.505***	1.100	10.343***	1.274	8.696***	0.944	3.050***	1.228	5.412***	1.044
Secondary school	11.822***	1.312	11.749***	1.481	11.932***	1.137	7.191***	1.432	4.678***	1.186
High school	9.548***	1.271	10.393***	1.451	9.978***	1.105	5.348***	1.380	4.520***	1.168
Community college	13.866***	1.661	14.437***	1.898	14.243***	1.454	8.101***	1.787	6.026***	1.510
College	10.533***	1.449	11.673***	1.660	11.086***	1.259	5.794***	1.579	5.157***	1.332
Wage Job	0.831	1.308	1.292	1.466	1.014	1.141	0.268	1.396	0.711	1.144
Employer	−1.016	1.559	2.937*	1.781	0.462	1.372	−2.627	1.665	2.837**	1.386
Job seekers	−1.941	1.593	0.524	1.828	−1.035	1.405	−2.430	1.699	1.245	1.419
Retired	3.912**	1.735	2.805	2.013	3.541***	1.525	2.932	1.874	0.659	1.582
Homemaker	−0.228	1.414	−0.585	1.617	−0.365	1.242	0.043	1.514	−0.384	1.259
Overweight	0.328	0.677	0.028	0.778	0.219	0.598	0.352	0.721	−0.115	0.603
Obese	3.484***	0.880	0.930	1.021	2.562***	0.783	3.408***	0.932	−0.700	0.789
Morbidly obese	3.122**	1.375	3.481**	1.523	3.294***	1.170	1.706	1.514	1.546	1.220
General health insurance	2.031**	1.003	0.617	1.161	1.522*	0.887	1.949*	1.071	−0.347	0.890
Private health insurance	1.302	1.633	2.361	1.817	1.716	1.419	0.249	1.747	1.391	1.420
Cycling	4.878***	1.203	0.601	1.409	3.323***	1.037	5.129***	0.347	−1.555	1.136
Walking 10–29 min	0.631	0.846	3.443***	0.987	1.698**	0.746	−1.050	0.918	2.561***	0.774
Walking 30–59 min	2.511***	0.906	5.899***	1.050	3.817***	0.793	−0.205	0.988	3.787***	0.829
Walking 1–2 h	2.187*	1.124	5.854***	1.290	3.594***	0.987	−0.544	1.208	3.886***	1.013
Walking > 2 h	3.849***	1.462	6.670***	1.744	4.957***	1.303	0.893	1.578	3.858***	1.364
Resting	0.727	0.591	2.053***	0.694	1.235***	0.523	−0.235	0.637	1.379***	0.542
Moderate physical job	1.558***	0.627	2.831***	0.711	2.056***	0.552	0.294	0.671	1.670***	0.551
Heavy physical job	2.494*	1.412	2.809*	1.640	2.642**	1.264	1.349	1.482	1.258	1.258
Low income	−10.566***	1.401	−18.514***	1.596	−13.684***	1.202	−2.349	1.564	−10.758***	1.295
High income	4.002***	1.307	10.865***	1.512	6.635***	1.168	−1.072	1.373	7.237***	1.165
Marmara	2.522**	1.191	0.686	1.393	1.860*	1.043	2.461*	1.301	−0.496	1.100
Aegean	−9.631***	1.604	−12.066***	1.849	−10.661***	1.386	−4.589***	1.773	−5.858***	1.487
Mediterranean	−10.398***	1.380	−8.603***	1.593	−9.843***	1.211	−7.207***	1.498	−2.694**	1.243
Black Sea	−16.595***	1.239	−24.505***	1.415	−19.769***	1.069	−6.016***	1.388	−13.143***	1.154
Central Anatolia	−0.500	1.266	−1.410	1.483	−0.849	1.115	0.161	1.374	−0.947	1.163
Southeastern Anatolia	−16.309***	1.773	−24.343***	2.027	−19.527***	1.548	−5.780***	1.934	−13.131***	1.606
*Continuous variables*										
Age	0.273***	0.028	0.255***	0.032	0.270***	0.024	0.175***	0.031	0.095***	0.026
Sports Time	1.205***	0.186	1.033***	0.213	1.155***	0.153	0.817***	0.218	0.342**	0.179
Tobacco	−7.332***	0.666	−2.829***	0.768	−5.721***	0.588	−6.730***	0.713	0.757	0.602
Alcohol	−0.282***	0.098	−0.167	0.114	−0.242***	0.087	−0.229**	0.103	−0.019	0.087
Number of children under 7	−0.120	0.463	−1.765***	0.532	−0.741*	0.404	0.766	0.504	−1.399***	0.419
Number of kids ages 7–14	−1.837***	0.405	−1.247***	0.467	−1.636***	0.352	−1.413***	0.444	−0.252	0.370
Number of adults	0.022	0.228	0.185	0.256	0.083	0.201	−0.070	0.240	0.142	0.197
Actual/Expected probability values	0.473/0.461	0.571/0.566	0.400/0.385	0.702/0.668	0.846/0.817					

ME shows marginal effects

***p < 0.01, **p < 0.05, *p < 0.10.

## Discussion and policy implications

The marginal effects derived under different probabilities show that individual socio-demographic, economic, and habitual factors significantly influence their F&V intake likelihood (Table 3). The marginal effects vary greatly as the calculated probability differs. First, we will focus on statistically significant dummy variables. Apart from fruit intake among individuals who consumed one or more servings of vegetables per day, in all other probabilities men consumed significantly fewer fruits and vegetables than women. Meanwhile, the probability of vegetable intake in the population (i.e. marginal probability, Prob(y_k_ = 1|x_ik_), where k = 1,2) is significantly lower among males than females (11.83%), and the probability of vegetable intake given the probability of one or more servings of fruit per day (7.42%) (i.e., the conditional probability). The joint probability of both foods (7.91%) is also lower among males in Turkey. The results show that the gender distinction in fruit and vegetable intake is obvious, as well as heterogeneity between the segments of the population. Men consume much fewer vegetables than women, which is followed by the probability of consuming two foods together and consuming vegetables among those who consume fruit. The result is consistent with the study of young American adults where men consumed approximately 8.5% less F&V than women [30]. Similarly, Malaysian men were 4.8% and 9.3%, respectively, less likely to consume fruits and vegetables per day than women [31]. A recent study confirmed that female-headed households consume more F&V [32]. In Turkey, being a male household head reduces the probability of monthly consumption of fresh or frozen vegetables by 2.4% and lowers the expenditures by 5.9 Turkish Lira (TL) in comparison to female household heads [12]. In general, the factors impacting health are expected to affect women more than men. For example, women who watch health television programs are likely to be more aware of the health benefits of F&V than men. Therefore, such findings may be attributed to men’s poorer nutritional knowledge and men being less likely to know the benefits associated with consuming the recommended intake. By expanding the scope and duration of health-related topics on television programs throughout the day, local and regional public health-related interventions may influence F&V consumption in the family. In a country like Turkey with a male-dominated workforce, policymakers at the national and local levels can increase male F&V consumption probability by urging employers to include a daily F&V option in lunches or promote F&V eating by offering them free or discounted prices in the workplace. Additionally, employers can ensure that vending machines include F&V options.

In all explored consumption likelihoods (i.e., different population segments), married individuals are more likely to consume fruits and vegetables than individuals with different marital statuses, and these individuals consume relatively more vegetables. A study in Uganda found that married participants were more likely to consume five or more servings of fruit and/or vegetables in a typical week [33]. Such a level of association has been supported by other studies [34]. Marriage and friendship form the basis of a regular meal pattern that includes fruits and vegetables and provides an opportunity for social interaction for greater food consumption. Such behaviors are explained by the theoretical model of social integration and social control associated with married couples’ efforts to control each other’s health behaviors [35]. In an average household of married couples, a spouse, especially a woman, keeps such foods at home to prepare a rich meal for themselves and their family. In empirical studies conducted in the United Kingdom [36], Canada [37], and Turkey [12], married households have been found to consume more F&V than households not married.

Education creates a set of social, behavioral, and psychological foundations that promote healthy lifestyles for individuals. A non-linear positive relationship was found between an individual’s education and F&V intake. The better educated choose to eat healthy produce, including F&V [12, 38, 39]. In all probabilities examined, secondary and university graduates are more likely to eat F&V. The effects of education on the probability of fruit intake among vegetable consumers and the probability of vegetable intake among fruit eaters were relatively lower than the other probabilistic effects from all education strata. Meanwhile, the probability of fruit intake among those who consume vegetables was found to be higher than the probability of intake of vegetables among those who consumed fruit. The fact that individuals who make vegetable consumption a part of their daily life pay the same attention to their daily fruit intake (along with their increasing education level) is proof that they may well be aware of the benefits of nutrition derived from fruits. We also found that the probability of consuming vegetables among fruit eaters was the lowest among all other probabilities as the individual’s education level increased. From the least educated to the most educated, it would be the right decision to switch to public programs that include the disease-fighting aspects of vegetables and their nutritional contribution to health.

The lesser educated, often unemployed or working lower-paying jobs and likely residing in suburbs face a greater risk of food insecurity or food budget constraints. In such cases, subsidies for F&V may be beneficial. F&V consumption should be encouraged for children two years old and older to assure they eat the recommended amount for reducing the risk of chronic diseases resulting from conditional such as vitamin deficiency. To this extent, the ministries of health and education can cooperate by adding topics addressing healthy nutrition to the school curricula and help mitigate household health expenditures while expanding the national healthcare system. Such coordinated efforts raise awareness and subsequently increase F&V consumption.

Compared with students and military service individuals, the probability of most F&V consumption increases among wage earners, though insignificant effects. Interestingly, among the employed, the probability of fruit consumption decreases for those who make vegetable consumption a part of their daily life, while the probability of consumption of vegetables increases significantly among fruit eaters. On the other hand, among job seekers, the probability of fruit consumption and the consumption of both foods together decrease. Interestingly, among the different layers of the explored population, fruit and vegetable consumption habits increase among the retired, while it decreases among homemakers, but in the case of the latter group, the results are statistically insignificant. Results show also that the employed may have decided that plant-based diets have become popular as a way to mitigate the diet’s environmental footprint and improve human health and animal welfare. The current study results coincide with Heo et al. [40] that employees had a low frequency of F&V consumption. In a study conducted in Ghana, the self-employed household head or working in the public sector had a reduced fresh F&V expenditure [41]. Terin et al. [12] reported that the consumption probabilities of fresh and frozen F&V were 2.5% and 1.78%, respectively, lower than that in the households of unemployed heads in Turkey. Although the current study does not identify the underlying reasons for such a consumption pattern, providing F&V as a snack in the workplace may increase fruit and vegetable intake for employees.

Obese and morbidly obese individuals tend to have higher F&V uptake in the distinct segments of the examined population. However, fruit consumption intake is statistically significant among obese individuals who make a habit of daily vegetable consumption. Meanwhile, the morbidly obese are more likely to have higher fruit intakes and are almost twice as likely to consume vegetables and both foods as compared to obese people. Tohill et al. [42] reported the F&V intake of individuals with a normal BMI was lower than the overweight or obese. Consuming fruit more than four times a week may have increased the risk of obesity [43]. In an Australian study, obese and overweight women were more likely to be in the highest quartile for combined fruit and vegetable intake and meet the “2 and 5” target or consume five or more servings of fruit and vegetables per day [44]. The energy supply due to F&V consumption of those with high BMI can be controlled by ensuring their participation in appropriate, regular physical activities along with dietary programs.

This study found that individuals with general health insurance are likely to increase their fruit intake, while those with private health insurance are more likely to consume vegetables both in the population (i.e., the marginal probability) and among fruit-consuming vegetable eaters (i.e., the conditional probability) but their effects found insignificant. F&V bill programs (also called bill of material programs, BoM,) can stimulate increased consumption of F&V among those living in poverty [45]. This simulation model typically allows a healthcare provider to “prescribe” eating fresh F&V to patients with diet-related chronic diseases while they receive nutritional guidance in a clinical setting. Such F&V bill programs have stimulated healthy eating habits [7, 45]. A study using private health insurance as a confounding factor in F&V intake in Australia showed a positive result, given that those with private health insurance tend to be highly educated and high-income individuals [46].

On the other hand, individuals who have the habit of cycling for at least ten minutes a day are more likely to consume one or more servings of fruit a day, consume more among those who eat both foods together, and are more likely to consume fruits among those who consume vegetables. Cyclists who experience energy loss due to exercise are expected to make up for the such loss by eating more fruit. Parallel to such a finding, compared with those with no or less than ten minutes of walking a day, the more time spent walking, the more likely individuals are to consume more fruit, vegetables, or both, and consume vegetables among daily fruit eaters. However, such an effect is more pronounced among those who make vegetable consumption a part of their daily life, almost twice as much as those who consume fruit.

A similar result exists among workaholics, where those who rest less than four hours a day are more likely to consume fruit and vegetables, while those who make their daily fruit intake a lifestyle increase their vegetable intake. This behavior increases the risk of unbalanced nutrition and severe health problems. Since those individuals tend to be highly educated, enjoy a relatively high income, and have regular medical checkups, they may be receptive to messages encouraging eating fruits and vegetables as snacks. Exercise and high consumption of F&V have a significant positive impact on the health of individuals [1].

Policies encouraging individuals to participate in various forms of physical exercise result in a healthier society and increase F&V consumption. Additionally, the creation of educational curricula that support the upbringing of healthier individuals (e.g., including courses that motivate individuals into participating in regular sports activities in school) would encourage individuals both to regularly exercise and consume healthier foods. On the other hand, as physical labor from moderate to vigorous intensity increases, individuals can meet their increased energy and other nutritional needs with fruit and vegetable intake. Heavy manual labor workers are relatively more likely to consume fruits and vegetables than lower manual labor workers, and they are also likely to consume more vegetables than fruits. Those who work jobs that are more physically demanding may replace some of their lost energy and nutritional needs with fruit and vegetables.

Individuals in the two extreme income quintiles, especially very poor families, have a very low probability of fruit and vegetable consumption in all segments of the population, while very wealthy families have a higher likelihood of fruit and vegetable intake compared to families in the middle-income bracket. Considering that very poor families spend almost all of their income on food, it is evident that they do not have access to sufficient nutritious food and are vulnerable to inadequate food consumption. The state could provide food and other services for low-income pregnant women and children up to a specific nutritional-risk age by developing a supplemental food program for women, infants, and children. On the other hand, when compared to families in the two extreme income brackets, we found that those with higher incomes made more fruits and vegetables a part of their daily lives, their vegetable intake outweighing fruit consumption. There is ample evidence in the literature confirming such a correlating relationship [11, 12].

The worldwide increase in family income and public health education in the last decade has contributed to the F&V consumption increase [38]. Affordability has been a reason for low-income households’ limited F&V consumption [47]. Yen et al. [31] reported that individuals in the lowest income group tend to consume fewer vegetables compared to those in the highest income category (e.g. individuals in the highest income group in England consumed more F&V [48]. Several other studies stressed that F&V consumption increased with income [12, 41]. In our study, as income increases, individuals prefer vegetable portions to fruit portions. Considering the nutritional values provided by different ranges of seasonal vegetables, it is expected that individuals will tend to prefer relatively more expensive vegetables to lower-priced vegetables as income increases.

Finally, in the examination of the link between regions (the last of the binary variables), where a person resided in the country and F&V consumption shows a positive relationship. As compared to individuals residing in the East Anatolia region, residents of Marmara consumed more F&V, whereas individuals residing in the Aegean, Mediterranean, Black Sea, and Southeastern Anatolia regions consumed fewer F&V. The result for Central Anatolians was insignificant. Compared to the reference region, individuals residing in the Black Sea region have very low fruit and vegetable intakes, while in regions with a confirmed statistically significant effect, the probability of individuals consuming fruits and vegetables is almost identical. In regions with low F&V consumption, encouraging more F&V consumption and the individual willingness to change eating habits is of great importance. Particularly, individuals should be motivated and encouraged to participate actively in lifestyle intervention programs (i.e., the Healthy Portions program) and to learn about the benefits of F&V through government-run public service advertisements in the media. Support programs encouraging rural populations can emphasize increased F&V consumption of raised produce.

Focusing on continuous variables, the increasing age of individuals increases the probability of F&V consumption in all segments of the population. However, the significant increase is in fruit intake, while the least uptake is in vegetable intake among fruit consumers. A plentiful intake of fruits and vegetables is important at any age, as such foods are the top source of fiber and other nutrients essential for good health. The intake of such nutrients should be made a habit early in life, as many of these nutrients help prevent or mitigate the risk of disease as it progresses over the years. Studies in Italy [49], the United Kingdom [50], and Malaysia [31] reported that an increase in the age of the household head increased fruit consumption. A similar relationship between the age of individuals and F&V consumption probability was obtained in studies conducted in Turkey [12] and South Korea [31]. Health literacy campaigns (e.g., creating public service ads, messages, advertisements, and social media content with private and public collaboration) regarding disease prevention and health maintenance by eating fruits and vegetables that are easy to understand can be implemented through mass media. Providing additional support for fruit and vegetable consumption among the young and middle-aged should be considered, such as the creation of mobile phone apps (e.g., businesses can help increase F&V intake by digitizing their messages regarding the availability and wholesomeness of F&V).

As time spent on playing sports increases, F&V intakes increase. F&V intake is likely to be higher because such activities require more energy. Meanwhile, tobacco and alcohol intake negatively affect both food intakes, but the effect of tobacco intake is both much more pronounced and far more potent than alcohol. Fruit and vegetable intakes are also affected by the appetite suppression properties of those two habits. It is particularly noteworthy that the probability of eating fruit by smokers is approximately 2.5 times lower than that of vegetables, suggesting that smoking may replace fruit snacking. Furthermore, the probability of reduced fruit consumption by smokers is higher among all considered factors. Yen et al. [31] found that the probability of fruit consumption by smokers was 6.72% lower than that of non-smokers. Daily smoking rates were less in groups with high F&V consumption compared to others [52]. A study of Canadian adults reported that those who never smoked, former smokers, and the elderly consumed more F&V than other groups [53].

The presence of children between 7 and 14 years old in the family significantly, although by a relatively small percent, lowers the probability of both fruit and vegetable consumption. Contrarilty, the increase in the number of adults in the household is likely to increase the intake of one or more servings of fruit per day but their impacts are insignificant. Adults with children may eat fewer fruits and vegetables as they are more likely to have to accommodate children who want to eat salty snacks, pizza, cheese, beef, ice cream, cake or cookies, high-fat foods such as sausage or processed meats, and nut-butters. F&V consumption can be increased in those households through creative business efforts. For example, parents’ preferences can be shaped by social media and electronic messages (e.g., Short Message Service (SMS), e-mail) containing information about F&V benefits. On the other hand, an increase in the number of adults in the family significantly increases the probability of vegetable intake and triggers the odds of consuming vegetables among those who consume fruit daily.

## Conclusion

The current study investigated the relationship between F&V intake decisions of family members eating one or more servings of fruits and vegetables a day and individual and family characteristics. This approach fills the gap by accounting for intra-family heterogeneity in cross-sectional studies of F&V consumption. Additionally, F&V consumption likelihoods often depend on simultaneous decisions. The fact that both the intra-family heterogeneity parameter and the relationship between the two eating decisions have significant and inverse effects indicates that the attitudes and decisions of each family can differ greatly. It is more reasonable to evaluate the results obtained the reflection of different characteristics of family members on the decision of distinct consumption likelihoods, rather than an extraordinary expectation. Studies that ignore the multilevel data structure and simultaneous fruit and vegetable consumption decisions are not expected to reach such counterfactual inferences.

Being male, regardless of job type, having an income, residing in Aegean, Mediterranean, Black Sea, and Southeastern Anatolia regions, smoking, alcohol consumption, and presence of children under age 7 and ages 7 to 14 in a household were factors associated with less likely F&V consumption. However, a positive influence on F&V intake decision was associated with marital status, education, BMI, general and private health insurance coverage, bicycling, walking, resting, performing physically demanding jobs, high income, residence in Marmara region, age, time spent on sports, and number of adults in the household. Elderly individuals consume more fruit, and fruit consumption is higher among those who also consume vegetables. The results show that increased fruit consumption is associated with more frequent vegetable consumption. Similarly, as an individual has more education, fruit intake among those who consume vegetables is higher than among those who consume only fruit. Considering the relationship between high education level and high income, it is clear that the priority of individuals in this group is high fruit intake despite relatively high prices. To increase F&V consumption and develop sustained F&V consumption, policies raising awareness of F&V benefits within the family are desirable in addition to efforts at the society level. Enhancing F&V consumption could lead to healthier current and future generations and possible health cost reduction.

The results suggest segmenting individuals by their socioeconomic, demographic, lifestyle, and income factors to develop and implement separate, segment-specific policies rather than attempting a one-size-fits-all policy. For example, there is a need to formulate and implement policy to increase F&V consumption among low- and middle-income households in Turkey. The elderly will benefit from information about the importance of vitamins in F&V in improving physical and mental health. State-run television channels can promote “healthy lifestyles” characterized by F&V consumption and affect housewives which then encourage family members to eat more F&V. Information about F&V can be conveyed to the illiterate population visually or verbally through social media via cell phones. Family-oriented programs should exploit the multi-faceted benefits of F&V by emphasizing the functional ingredients of fruits and vegetables and targeting specific segments of the population such as smokers. A separate path promoting F&V consumption can be fruit and vegetable snacks accessible for workers at places of employment, stressing the link between health maintenance and productivity.

Children need to be a target of programs tailored to various age groups and possibly genders. Parents need to be educated about the importance of F&V intake by children beginning from an early age. Public service announcements using age-appropriate visual, printed, and broadcast media to raise healthier future generations as well as to prevent and reduce serious health problems that may arise later in life will help reach such goals. Hiring dietitians in family healthcare centers to offer vegetable and fruit diet guidelines should be considered to mitigate diseases that may result from insufficient F&V consumption. Efforts promoting F&V eating could be supplemented by the construction of sports fields, walking areas, and bicycle paths to encourage physical activity in residential neighborhoods leading to a healthier quality of life. Innovative interventions aiming at increasing F&V uptake should be a continuing focus in Turkey. Meanwhile, the differences in F&V intake across regions suggest that interventions to reach the recommended level of consumption should involve programs recognizing the regional specificity of low F&V intake.

The study has several limitations. The use of cross-sectional data limits the causal analysis of the decision to eat F&V. Multi-year panel data, if available, could permit a deeper analysis and provide knowledge for additional detailed actions encouraging F&V eating. A richer dataset will also allow the application of a different modeling framework overcoming the limitations of cross-sectional data use in the estimation of a latent dependent variable model. Also, the results obtained for Turkey, an emerging economy with a fast-growing population, may not be readily transferrable to every country. However, the results of the current study provide insights that can serve as a benchmark for assessing similar topics in other countries. Finally, the focus on the consumption of fruits and vegetables as the source of eliminating or reducing any vitamin and micronutrient deficiency in the current study implicitly recognizes that such deficiencies can have also other reasons such as the unaccounted household behavior including cooking and preparation methods of frhes produce, household storage methods of, or purchase of prepared or processed fruits and vegetables. Additional data could be collected in the future to examine such causes.

## Data Availability

Datasets used in the current study are available from the corresponding author upon reasonable request.

## Appendix

See Tables 4, 5, 6 and 7.

**Table 4:** Vitamin and micronutrient content of commonly eaten fruits in Turkey

Fruit	Vitamin C (mg)	Vitamin D (IU)	Vitamin E (mg)	Vitamin K (mcg)	Vitamin A (IU)	VitaminB12 (mcg)	Vitamin B9 (Folic acid) (mcg)	Vitamin B6 (mg)	Iron (mg)	Potassium (mg)	Zinc (mg)	Magnesium (mg)	Fiber (g)
Oranges	71.0	0	0.0	0.0	250.0	0.0	30.0	0.1	0.8	196.0	0.10	10.7	4.5
Watermelon	8.1	0	0.1	0.1	569.0	0.0	3.0	0.0	0.2	112.0	0.10	10.0	0.4
Apples	4.6	0	0.2	2.2	3.0	0.0	3.0	0.0	< 0.1	95.0	0.02	4.7	2.0
Cranberries	13.3	0	1.2	5.1	60.0	0.0	1.0	0.1	0.3	85.0	0.10	0.4	4.6
Blueberries	9.7	0	0.6	19.3	54.0	0.0	6.0	0.1	0.3	77.0	0.20	6.0	2.4
Tangerines	26.7	0	0.2	0.0	681.0	0.0	16.0	0.1	0.2	166.0	0.10	12.0	1.8
Mandarins	26.7	0	0.2	0.0	681.0	0.0	16.0	0.1	0.2	166.0	0.10	12.0	1.8
Banana	8.7	0	0.1	0.5	64.0	0.0	20.0	0.4	0.3	358.0	0.20	27.0	2.6

Amounts per 100 g

*Source*: https://nutritiondata.self.com/facts/fruits-and-fruit-juices/1970/2#ixzz7OSYTIOBK

*Source*: http://www.dietandfitnesstoday.com/vitamins-and-minerals.php

**Table 5:** Vitamin and micronutrient content of commonly eaten vegetables in Turkey

Vegetable	Vitamin C (mg)	Vitamin D (IU)	Vitamin E (mg)	Vitamin K (mcg)	Vitamin A (IU)	Vitamin B12 (mcg)	Folic acid (mcg)	Vitamin B6 (mg)	Iron (mg)	Potassium (mg)	Zinc (mg)	Magnesium (mg)	Fiber (g)
Tomato	16.0	0.0	0.5	7.9	1496.0	0.0	68.0	0.1	0.5	212.0	0.1	8.0	0.9
Yellow Onion	7.4	0.0	0.0	0.4	2.0	0.0	19.0	0.1	0.2	146.0	0.2	10.0	1.7
Green Pepper	80.4	0.0	0.4	7.4	370.0	0.0	10.0	0.2	0.3	175.0	0.1	10.0	1.7
Eggplant	2.2	0.0	0.3	3.5	27.0	0.0	22.0	0.1	0.2	230.0	0.2	14.0	3.4
Cucumber	2.8	0.0	0.0	16.4	105.0	0.0	14.0	0.0	0.3	147.0	0.2	13.0	0.5

Amounts per 100 g

Source: https://nutritiondata.self.com/facts/fruits-and-fruit-juices/1970/2#ixzz7OSYTIOBK

Source: http://www.dietandfitnesstoday.com/vitamins-and-minerals.php

**Table 6:** Maximum likelihood estimates from the bivariate probit model

Variables	Fruits		Vegetables	
	Coefficient	Standard Error	Coefficient	Standard Error
Constant	−0.654***	0.088	−0.315***	0.088
*Discrete variables*				
Gender	−0.129***	0.027	−0.256***	0.027
Married	0.057**	0.025	0.082***	0.025
Elementary school	0.289***	0.036	0.327***	0.036
Secondary school	0.406***	0.043	0.367***	0.044
High school	0.376***	0.043	0.366***	0.043
Community college	0.496***	0.057	0.477***	0.057
College	0.437***	0.049	0.413***	0.049
Wage Job	−0.038	0.046	−0.057	0.046
Employer	−0.056	0.055	0.017	0.054
Job seekers	−0.130**	0.054	−0.110**	0.053
Retired	0.038	0.060	−0.038	0.060
Homemaker	−0.036	0.049	−0.075	0.049
Overweight	0.048**	0.024	0.066***	0.024
Obese	0.116***	0.030	0.091***	0.031
Morbidly obese	0.110**	0.046	0.177***	0.047
General health insurance	0.041	0.035	−0.014	0.036
Private health insurance	0.067	0.057	0.096*	0.057
Cycling	0.147***	0.044	0.032	0.044
Walking 10–29 min	−0.075**	0.029	−0.007	0.030
Walking 30–59 min	0.018	0.031	0.096***	0.032
Walking 1–2 h	−0.034	0.039	0.058	0.039
Walking > 2 h	0.054	0.051	0.091*	0.051
Resting	0.033	0.021	0.072***	0.021
Moderate physical job	0.072***	0.022	0.109***	0.022
Heavy physical job	0.060	0.051	0.097*	0.051
Low income	−0.246***	0.047	−0.328***	0.048
High income	0.058	0.047	0.171***	0.047
Marmara	0.088**	0.041	0.036	0.041
Aegean	−0.183***	0.055	−0.201***	0.055
Mediterranean	−0.229***	0.048	−0.106**	0.048
Black Sea	−0.389***	0.041	−0.453***	0.041
Central Anatolia	0.032	0.044	−0.019	0.044
Southeastern Anatolia	−0.375***	0.060	−0.396***	0.060
*Continuous variables*				
Age	0.009***	0.001	0.007***	0.001
Sports Time	0.034***	0.007	0.028***	0.007
Tobacco	−0.222***	0.023	−0.067***	0.024
Alcohol	−0.008**	0.004	−0.002	0.004
Number of children under 7	−0.009	0.016	−0.038**	0.016
Number of kids ages 7–14	−0.026*	0.014	−0.001	0.014
Number of adults	0.004	0.008	0.005	0.008
τ	0.733***	0.007	–	–

**p* < 0.10, ***p* < 0.05, ****p* < 0.01

**Table 7:** Marginal effects of explanatory variables on eating one or more servings of fruits and vegetables in Turkey using the bivariate probit model

Variables	Prob (y1 = 1)		Prob (y2 = 1)		Prob (y1 = 1, y2 = 1)		Prob (y1 = 1|y2 = 1)		Prob (y2 = 1|y1 = 1)	
	ME*100	Std. Err	ME*100	Std. Err	ME*100	Std. Err	ME*100	Std. Err	ME*100	Std. Err
*Discrete variables*										
Gender	−5.121***	1.084	−10.087***	1.075	−7.065***	0.961	−0.186	1.043	−5.981***	0.763
Married	2.274**	1.011	3.235***	0.998	2.663***	0.888	0.766	0.982	1.636**	0.713
Elementary school	11.467***	1.431	12.886***	1.417	12.176***	1.226	5.744***	1.467	5.442***	1.081
Secondary school	16.118***	1.739	14.444***	1.725	15.650***	1.511	10.088***	1.742	4.641***	1.279
High school	14.934***	1.709	14.422***	1.685	14.896***	1.484	8.776***	1.705	5.152***	1.247
Community college	19.704***	2.269	18.778***	2.242	19.558***	1.982	11.716***	2.238	6.592***	1.636
College	17.344***	1.960	16.274***	1.942	17.119***	1.713	10.454***	1.937	5.593***	1.421
Wage Job	−1.494	1.833	−2.253	1.792	−1.799	1.611	−0.433	1.766	−1.180	1.268
Employer	−2.207	2.166	0.683	2.137	−1.130	1.892	−2.842	2.129	1.546	1.546
Job seekers	−5.154***	2.154	−4.340**	2.103	−4.898***	1.895	−3.378*	2.072	−1.256	1.486
Retired	−1.502	2.382	−1.510	2.357	0.371	2.097	2.508	2.308	−1.908	1.678
Homemaker	−1.425	1.963	−2.932	1.925	−2.014	1.728	0.017	1.890	−1.767	1.361
Overweight	1.917**	0.934	2.583***	0.926	2.190***	0.824	0.725	0.905	1.261**	0.658
Obese	4.599***	1.220	3.568***	1.212	4.255***	1.082	3.182***	1.169	0.871	0.852
Morbidly obese	4.358***	1.845	6.953***	1.841	5.392***	1.608	1.055	1.837	3.753***	1.350
General health insurance	1.617	1.400	−0.547	1.394	0.810	1.232	2.108	1.364	−1.171	0.998
Private health insurance	2.660	2.280	3.791*	2.263	3.118	2.028	0.892	2.166	1.920	1.575
Cycling	5.820***	1.751	1.269	1.731	4.149***	1.524	5.809***	1.737	−1.560	1.265
Walking 10–29 min	−2.965***	1.169	−0.266	1.169	−1.969**	1.022	−3.169***	1.158	1.106	0.853
Walking 30–59 min	0.731	1.253	3.794***	1.258	1.904*	1.101	−1.265	1.234	2.783***	0.912
Walking 1–2 h	−1.338	1.532	2.286	1.526	0.027	1.343	−2.750*	1.506	2.471**	1.104
Walking > 2 h	2.159	2.006	3.580*	2.020	2.723	1.780	0.448	1.935	1.970	1.431
Resting	1.315	0.843	2.846***	0.842	1.911***	0.747	−0.093	0.811	1.746***	0.596
Moderate physical job	2.849***	0.884	4.306***	0.878	3.434***	0.783	0.821	0.849	2.257***	0.620
Heavy physical job	2.395	2.012	3.812**	2.013	2.959*	1.794	0.584	1.911	2.055	1.402
Low income	−9.766***	1.867	−12.923***	1.876	−11.070***	1.575	−3.822**	1.982	−6.231***	1.480
High income	2.313	1.870	6.750***	1.854	4.026***	1.685	−1.119	1.721	4.499***	1.248
Marmara	3.489**	1.626	1.433	1.632	2.743**	1.407	3.113**	1.647	−0.383	1.219
Aegean	−7.256***	2.175	−7.911***	2.174	−7.582***	1.844	−3.768*	2.277	−3.243**	1.687
Mediterranean	−9.103***	1.896	−4.183**	1.894	−7.326***	1.662	−7.879***	1.864	0.636	1.369
Black Sea	−15.469***	1.637	−17.860***	1.630	−16.541***	1.401	−7.487***	1.687	−7.731***	1.247
Central Anatolia	1.267	1.738	−0.760	1.746	0.509	1.514	1.833	1.740	−1.189	1.287
Southeastern Anatolia	−14.876***	2.397	−15.610***	2.342	−15.311***	2.071	−8.059***	2.394	−6.152***	1.728
*Continuous variables*										
Age	0.348***	0.039	0.272***	0.038	0.323***	0.034	0.240***	0.038	0.068***	0.028
Sports Time	1.349***	0.260	1.085***	0.278	1.263***	0.225	0.912***	0.275	0.287	0.216
Tobacco	−8.801***	0.924	−2.652***	0.926	−6.553***	0.820	−8.382***	0.890	1.757***	0.655
Alcohol	−0.324**	0.140	−0.060	0.140	−0.227*	0.126	−0.329***	0.131	0.095	0.095
Number of children under 7	0.342	0.630	−1.488***	0.623	−0.351	0.549	1.199**	0.626	−1.372***	0.456
Number of kids ages 7–14	−1.039*	0.551	0.034	0.540	−0.641	0.475	−1.180**	0.556	0.492	0.402
Number of adults	0.155	0.317	0.199	0.311	0.173	0.280	0.063	0.304	0.094	0.219

ME shows marginal effects

**p* < 0.10, ***p* < 0.05, ****p* < 0.01

## Abbreviations

THS: Turkish Health Survey
TSI: Turkish Statistical Institute
F&V: Fruits and vegetable
NCDs: Non-communicable diseases
LFVI: Low fruit and vegetable intake
BP: Blood pressure
SOEU: Statistical Office of the European Union
NUTS: Nomenclature of Territorial Units for Statistics
VIF: Variance inflation factor
LR: Likelihood Ratio
LM: Lagrangian Multiplier
BMI: Body mass index
SMS: Short Message Service

## Footnotes

1 Against the possibility of an endogeneity problem caused by depression in the system, the depression variable was run against a number of variables and then its residuals were calculated and used together in the probability of fruit and vegetable consumption. The insignificance of the variable confirms that there is no such endogeneity problem in the system.

2 Since the data are on a national basis, representative weights were used in parameter estimation, thanks to a referee’s warning.

3 All analyses were performed using NLOGIT-6 statistics software.

4 Bivariate probit model and marginal effect results are given in the appendix of Tables 6 and 7, respectively.
